# Association between Present Teeth and Muscle Strength in Older People in Korea

**DOI:** 10.3390/jpm12071163

**Published:** 2022-07-18

**Authors:** Ji-Eun Kim, Na-Yeong Kim, Choong-Ho Choi, Ki-Ho Chung

**Affiliations:** 1 Department of Preventive and Public Health Dentistry, Chonnam National University School of Dentistry, Gwangju 61186, Korea; angel761@jnu.ac.kr (J.-E.K.); 216622@jnu.ac.kr (N.-Y.K.); hochoi@jnu.ac.kr (C.-H.C.); 2 Dental Science Research Institute, Chonnam National University, Gwangju 61186, Korea

**Keywords:** aged, muscle strength, sarcopenia, tooth

## Abstract

Background: As the world population continues to age, interest in muscle strength loss in older people is increasing. This study aimed to confirm the association between present teeth and muscle strength in older people in Korea. Methods: Using data extracted from the 2014–2019 Korea National Health and Nutrition Examination Survey, we analyzed 5136 older people aged 65–79 years. The present teeth were based on 20 teeth, which is the criteria for comfortable mastication. The association of the risk of low muscle strength according to the present teeth was assessed using multiple logistic regression analysis, and the association was confirmed by dividing into subgroups according to sex. Results: The prevalence of low muscle strength was 17.87% among all participants. Multiple logistic regression analysis confirmed the association between low muscle strength and present teeth; a significant association was found even in the model in which all covariates were adjusted (odds ratios (OR) = 1.35; 95% confidence interval (CI): 1.13–1.61). Subgroup analysis revealed a significant association between present teeth and low muscle strength even in the model in which both covariates were adjusted for sex (Men, OR = 1.41; 95% CI: 1.02–1.95; Women, OR = 1.31; 95% CI: 1.06–1.6). Conclusion: An association between present teeth and low muscle strength was confirmed in older people in Korea. These results indicate that the importance of oral hygiene management should be emphasized to prevent muscle strength loss in older people.

## 1. Introduction

As the global population continues to age, it is predicted that more than 20% of the world’s population will be over 60 years old by 2050 [1]. Aging is one of the common aspects among people who are socio-economically vulnerable, alongside poverty, low education, and unemployment, and older people are a category that deserves much attention in national prevention strategies [2]. As such, interest in the health of older people is increasing, and in particular, many related studies on muscle strength have been conducted [3,4,5,6,7,8]. Muscle strength is an important factor for maintaining vitality, mobility, and physical function in old age [3], and muscle strength loss is an independent risk factor for high mortality in older people [4].

There are several methods for measuring muscle strength, such as handgrip strength, leg muscle strength, and sit-to-stand [9]. In particular, handgrip strength is an inexpensive and simple factor for measuring strength [10], and several studies have used handgrip strength as an indicator of strength in individuals [5,11,12]. Bohannon reported that handgrip strength has been established as an indicator of muscle condition, especially in older people [5]. Recently, the 2019 Asian Working Group recommended using handgrip strength as an indicator of muscle strength to diagnose sarcopenia [11]. It has been reported that handgrip strength is associated with future fracture risk [13] and may be a risk indicator for poor cognitive outcomes [7]. In addition, it was shown that handgrip strength was considerably associated with quality of life after hip fracture surgery in the elderly [14]. Therefore, low handgrip strength is further linked to functional, psychological, and social health domains [12] and can be used as an indicator to reflect overall health.

The oral cavity is closely related to overall health [15]. Oral health can be evaluated using several indicators; in particular, present teeth is used as a representative indicator of oral health in several studies [16,17,18,19]. A threshold of 20 teeth is a reasonable standard to indicate oral health [17] and is a predictive factor for mortality in Swedish [18] and Japanese [19] older people.

A previous study reported the association between oral health and muscle strength [20,21,22]. Although one study previously showed that present teeth were negatively correlated with low levels of handgrip strength and the possibility of sarcopenia after adjusting for all covariates, this study was conducted with a relatively small number of participants (600) [20]. Another study reported that low levels of handgrip strength in older men were associated with full denture use and less present teeth; however, oral-related adjustment covariates were limited [21]. In addition, each study reported results using various criteria for muscle strength [20,21,22].

This study attempted to evaluate the association between present teeth and handgrip strength using the latest standard of low muscle strength by adjusting various covariates for older people in a large sample representative of the Korean population. Therefore, this study aimed to evaluate the association between present teeth and muscle strength in older Koreans aged 65–79 years, and the null hypothesis was established: that there is no association between present teeth and muscle strength in older Koreans aged 65–79 years.

## 2. Materials and Methods

### 2.1. Study Population

Data were extracted from the 6th (2014–2015), 7th (2016–2018), and 8th (2019) Korea National Health and Nutrition Examination Survey (KNHANES VI-2,3 VII VIII-1), which is a cross-sectional survey. KNHANES is a survey based on a nationwide non-institutionalized Korean sample conducted by Korea’s centers for disease control and prevention.

**Figure 1:**
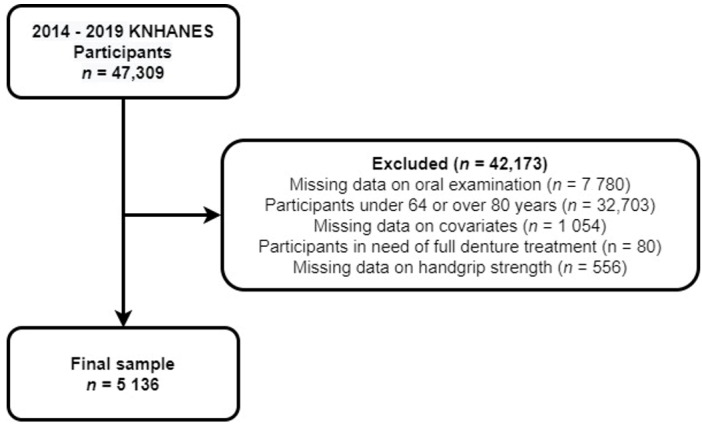
Flow chart of the selection process for the study population.

Among 47,309 participants, those who did not undergo oral and handgrip strength tests and those with missing covariates were excluded. For the grip strength test, subjects to be measured were selected according to the pre-examination examination and questionnaire (surgery history, pain, subjective survey participation, etc.). Finally, among older participants aged 65–79 years, those who were determined to require total denture treatment were excluded, and the data of 5136 individuals (men, 2338; women, 2798) were included in the final sample (Figure 1).

The data used in this study were used with the approval of the research ethics review committee of the Korea Disease Control and Prevention Agency (2013-12EXP-03-5C, 2018-01-03-P-A, 2018-01-03-C-A). Written informed consent was obtained from all participants before the investigation.

### 2.2. Muscle Strength

The Asian Working Group’s 2019 standards [11] for handgrip strength were applied. Low muscle strength was defined as handgrip strength <28 kg for men and <18 kg for women.

Using a digital handgrip strength dynamometer (digital grip strength dynamometer, T.K.K 5401, Japan), handgrip strength was measured in both hands three times in a standing position, and the maximum value was used.

### 2.3. Present Teeth

A dentist trained according to the KNHANES oral examination guidelines [23] performed oral examinations for participants and recorded the results. The present teeth were obtained as the sum of teeth existing orally among a total of 28 teeth, excluding the third molar. Participants were classified into two groups based on the results as follows: <20 teeth and ≥20 teeth.

### 2.4. Covariates

Data on the general characteristics of the participants, including sex, age, education level (≤primary school, middle, high, ≥college), and household income (lowest quartile, lower-middle quartile, upper-middle quartile, highest quartile), were collected.

We collected data on the general health status variables, including smoking history (nonsmoker, past smoker, current smoker), drinking alcohol (nondrinking, 1 time per month, ≥2 times per month), exercise (whether the participants practiced moderate-intensity physical activity for 2 h 30 min or more, high-intensity physical activity for 1 h 15 min or more, or a mix of moderate-intensity and high-intensity physical activity per week), body mass index (BMI, weight/height^2^), and comorbidities (number of cases diagnosed by a doctor for chronic diseases such as high blood pressure, diabetes, stroke, myocardial infarction or angina pectoris, arthritis, and cancer).

Oral health behavioral variables assessed were frequency of teeth brushing per day, use of oral hygiene products (sum of use of dental floss, interdental toothbrush, mouthwash, electric toothbrush, and other oral hygiene products), chewing problem, dental visits during the past year, and self-perceived oral health.

### 2.5. Statistical Analyses

KNHANES conducted data analysis by considering strata variables, cluster variables, and weights owing to the complex sample design as a complex sampling survey.

To assess sociodemographic characteristics of participants, the t-test or chi-square test was used by classifying the participants according to their handgrip strength and sex.

The association between the present teeth and the risk of low muscle strength was evaluated using logistic regression analysis. We further constructed four multiple regression models to identify potential covariates. Thereafter, the association was confirmed by dividing the participants into subgroups according to sex.

Statistical significance was set as a *p*-value < 0.05. The SAS 9.4 program (SAS Institute, Cary, NC, USA) was used for statistical analysis.

## 3. Results

### 3.1. Participants Characteristics

The average age of the participants was 71.20 ± 0.08 years (men:women = 70.98 ± 0.10 years:71.38 ± 0.10 years). A statistically significant difference was found between the sexes for all covariates, except for chewing problem and self-perceived oral health variables. The average present teeth in the participants was 19.01 ± 0.15, and men (18.46 ± 0.22) had fewer teeth than the average (Table 1).

### 3.2. Distribution of Participants According to Muscle Strength and Present Teeth

**Figure 2:**
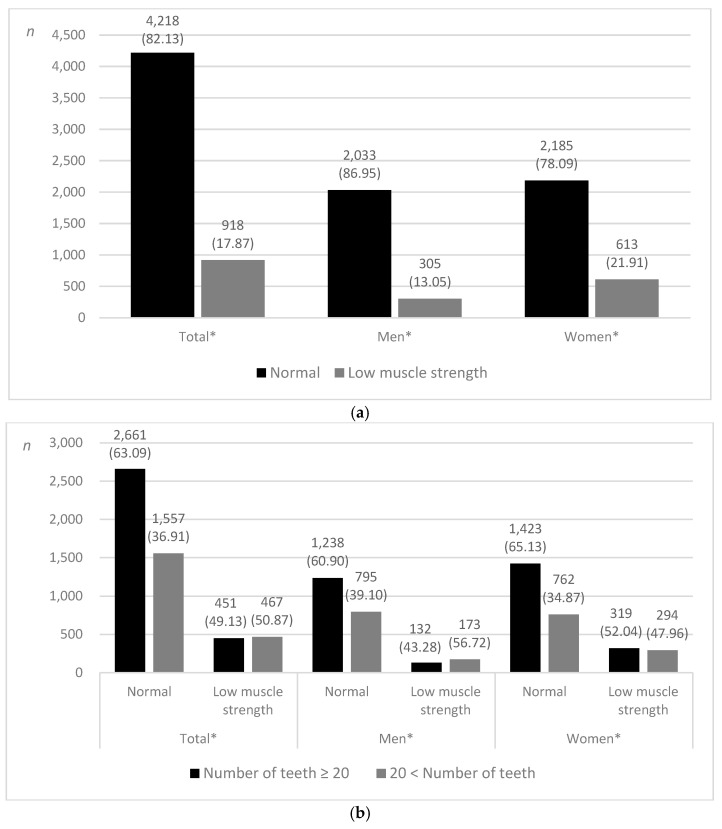
Distribution of participants. (**a**) According to the prevalence of low muscle strength. (**b**) Low muscle strength and normal muscle strength according to the present teeth, *n* (weighted %). * *p* < 0.001 using the chi-square test.

Among 5136 participants, 918 (17.87%) were classified as having low muscle strength. The prevalence of low muscle strength was higher among women (21.91%) than among men (13.05%) (Figure 2a).

Among all participants, more than half (50.87%) of those classified as having low muscle strength had fewer than 20 teeth, and the same distribution was observed in men (Figure 2b).

### 3.3. The Association between the Present Teeth and Low Muscle Strength

Multiple logistic regression analyses of the association of low muscle strength according to the present teeth (Model 1) revealed that individuals with less than 20 teeth had a higher risk of low muscle strength than those with more than 20 teeth (odds ratio (OR) = 1.73; 95% confidence interval (CI): 1.48–2.03). This association was also observed in model 2, which was adjusted for sex and age (OR = 1.48; 95% CI: 1.25–1.74); model 3, which was adjusted for general characteristics and general health status (OR = 1.36; 95% CI: 1.15–1.62); and model 4, which was adjusted for oral health behavior and status variables (OR = 1.35; 95% CI: 1.13–1.61) (Table 2).

Subgroup analysis of the participants by sex confirmed the association between present teeth and low muscle strength, as both the men and women subgroups were statistically significant in all models (*p* < 0.05). OR was higher in men than in women in all models (Model 1, men:women = 1.96 (1.47–2.62):1.71 (1.40–2.09); Model 2, men:women = 1.64 (1.21–2.21):1.40 (1.13–1.72); Model 3, men:women = 1.54 (1.12–2.12):1.29 (1.04–1.59); Model 4, men:women = 1.41 (1.02–1.95):1.31 (1.06–1.61), Table 3).

## 4. Discussion

Our study used data from a cohort representative of the Korean population and confirmed the association between present teeth and low muscle strength in older people in Korea. Considering the reports using various existing standards for muscle strength [20,21,22], the results of this study are meaningful in that they confirmed the association between present teeth and muscle strength using the latest standards.

Handgrip strength showed a significant positive association between overall strength and present teeth in the older population even after adjusting for covariates [6]. We used handgrip strength, measured in a standing position, to determine strength because this reflects both upper and lower extremity strength [24]. In this study, it is thought that the overall muscle strength of the body of older people was reflected by the handgrip strength using this measurement method.

A total of greater than 20 teeth enables functionally comfortable mastication [25], and masticating ability is strongly related to an individual’s nutritional status and quality of life [26]. In addition, based on a previous study in which 20 teeth were used as an important indicator to monitor oral health in older people [17], the criteria for the present teeth in this study were divided into ≥20 and <20 teeth.

In a previous study [27], a negative association between muscle mass and age was reported. The data used in our study are recorded as 80 years of age for participants over 80 years of age during the collection process. If participants over 80 years of age are included, it is considered that it will not be accurate when adjusting age as a covariate. In addition, it is a well-known phenomenon that edentulous patients have poor nutritional status [28], while muscle weakness is triggered by malnutrition [29]. Based on the aforementioned information, the age of the participants was limited to 65–79 years, and those in need of total denture treatment were excluded.

Autism spectrum syndrome (ASD) [30] and dementia [31] can affect oral health and/or handgrip strength. Since the KNHANSE data we used are derived from an interview or self-report method, it is thought that ASD, which lacks social communication, would have been excluded. Before measuring the handgrip strength, if there was any difficulty in measuring, an examination and questionnaire were conducted to exclude the participant. Therefore, it is thought that there would be no confounding effect due to dementia.

“Possible sarcopenia” was recently introduced as a screening index for sarcopenia to facilitate primary health care in the community [11]. In our study, low muscle strength was classified according to the above concept. In this study, the proportion of participants classified as having low muscle strength, which is defined as having possible sarcopenia, was 17.87%. Individuals with low muscle strength are at a high risk of developing sarcopenia, and lifestyle modifications, such as improved diet and exercise, would be beneficial to minimize this risk.

In the present study, multiple logistic regression analysis confirmed the association between present teeth and muscle strength as follows: the risk of low muscle strength was 1.73 (95% CI: 1.48–2.03) for those with fewer than 20 teeth compared to those with more than 20 teeth in the unadjusted model. In a study of Chinese adults [32], there was no association between tooth loss and handgrip strength in people over 60 years of age. However, in another study, it was reported that sarcopenia as a diagnostic criterion for handgrip strength and present teeth in participants aged over 80 years were related before adjusted [33]. Another study also reported an association between present teeth and low relative handgrip strength as continuous and categorical variables in adults [34]. This is in agreement with the results of our study, which suggests that oral health status, defined as having less than 20 teeth, may increase the risk of low muscle strength. Previous studies have reported that there is an association between oral health behaviors evaluated based on the frequency of brushing and the use of secondary oral care products and handgrip strength [35]. It is confirmed that the association is maintained in Model 4 (OR = 1.35; CI: 1.13–1.61), which was adjusted with the above-mentioned oral health behavioral variables, dental visits during the past year, and self-perceived oral health as covariates.

In addition, analysis of the participants after subgrouping by sex revealed that the present teeth and muscle strength were significantly related in both sexes, even when all covariates were adjusted (*p* < 0.05). In this model, the OR (1.41; 95% CI: 1.02–1.95) was higher in men than in women (OR = 1.31; 95% CI: 1.06–1.61). In a previous study [36], women had a higher prevalence of lower handgrip strength than men before 60 years of age, but men after 60 years of age had significantly increased prevalence of lower handgrip strength than women. This suggests that men have a higher risk of experiencing decreased handgrip strength as they age compared with women. In this study, present teeth is considered to be a variable that can indirectly represent the pattern of muscle strength reduction according to sex.

The mechanisms that could explain the association between present teeth and muscle strength are unknown. However, a previous study [37] reported a strong association between tooth loss and muscle mass thickness with respect to major masticatory muscles. In addition, chewing discomfort owing to tooth loss can affect nutritional status owing to improper eating habits [25]. Therefore, it is thought that present teeth affect the chewing and intake of food and may thereby indirectly affect whole-body muscle strength. Another potential mechanism is muscle strength and tooth loss owing to past or current inflammation [38]; interleukin-6 and tumor necrosis factor-α are common inflammatory markers, which are associated with present teeth and muscle strength [39,40], and high levels of these inflammatory markers may lead to loss of teeth and muscle strength.

Our study had several limitations. First, unfortunately, we could not confirm a causal association between the two investigated factors owing to the cross-sectional design of the study. Second, as we analyzed only secondary data in this study, information regarding periodontal-related variables was incomplete and could not be considered. Future longitudinal studies considering periodontal-related variables should be conducted to confirm the causal association between these two factors. Third, in the 7th KNHANES, the number of participants who received health surveys but did not take oral exams was relatively high due to limited research support from public health dentists, which appears to be a limitation of the data. Fourth, consideration for partial dentures is insufficient. In KNHANSE data, only the presence or absence of partial dentures or the need for partial denture treatment can be checked. Based on previous findings that low posterior occlusion is associated with a risk of low handgrip strength [41], it should be considered that the position of missing teeth and partial dentures could potentially affect handgrip strength.

Despite these limitations, to the best of our knowledge, this study is the first to identify the proportion of people with possible sarcopenia status in the Korean community in more than 5100 older people included from a study sample representative of the Korean population. In addition, this study is meaningful as it confirmed the association between oral health and muscle strength using two simple variables (present teeth and handgrip strength). Therefore, our results reject null hypothesis, and it is considered that there is a relationship between teeth and muscle strength in older Koreans aged 65–79 years.

## 5. Conclusions

The association between present teeth and low muscle strength was confirmed in older Koreans, and the importance of oral hygiene management should be emphasized to prevent muscle strength loss in older people.

## Figures and Tables

**Table 1:** General characteristics of the participants.

Characteristics	Total (*n* = 5136)	Men (*n* = 2338)	Women (*n* = 2798)	*p*-Value *
**Age, years**	71.20 ± 0.08	70.98 ± 0.10	71.38 ± 0.10	0.0023
**Education level**				
≤Primary school	2813 (54.77)	877 (37.51)	1936 (69.19)	<0.0001
Middle	842 (16.39)	460 (19.67)	382 (13.65)	
High	954 (18.57)	615 (26.30)	339 (12.12)	
≥College	527 (10.26)	386 (16.51)	141 (5.04)	
**Household income**				
Lowest quartile	2201 (42.85)	844 (36.10)	1357 (48.50)	<0.0001
Lower-middle quartile	1540 (29.98)	769 (32.89)	771 (27.56)	
Upper-middle quartile	842 (16.39)	426 (18.22)	416 (14.87)	
Highest quartile	553 (10.77)	299 (12.79)	254 (9.08)	
**Smoking**				
Nonsmoker	3147 (61.27)	487 (20.83)	2660 (95.07)	<0.0001
Past smoker	1488 (28.97)	1406 (60.14)	82 (2.93)	
Current smoker	501 (9.75)	445 (19.03)	56 (2.00)	
**Drinking alcohol**				
Nondrinking	2325 (45.27)	682 (29.17)	1643 (58.72)	<0.0001
1 time per month	1219 (23.73)	410 (17.54)	809 (28.91)	
≥2 times per month	1592 (31.00)	1246 (53.29)	346 (12.37)	
**Exercise**				
No	3324 (64.72)	1367 (58.47)	1957 (69.94)	<0.0001
Yes	841 (16.37)	971 (41.53)	841 (30.06)	
**BMI**				
<18.5 kg/m^2^	107 (2.08)	63 (2.69)	44 (1.57)	0.0001
Between 18.5 and 25 kg/m^2^	3074 (59.85)	1463 (62.57)	1611 (57.58)	
≤25 kg/m^2^	1955 (38.06)	812 (34.73)	1143 (40.85)	
**Comorbidities**				
0	1288 (25.08)	716 (30.62)	572 (20.44)	<0.0001
1	1960 (38.16)	939 (40.16)	1021 (36.49)	
≤2	1888 (36.76)	683 (29.21)	1205 (43.07)	
**Frequency of brushing teeth per day**				
≤1	943 (18.36)	569 (24.34)	374 (13.37)	<0.0001
2	2241 (43.63)	866 (37.04)	1375 (49.14)	
≤3	1952 (38.01)	903 (38.62)	1049 (37.49)	
**Use of oral hygiene products**				
0	3111 (60.57)	1471 (62.92)	1640 (58.61)	0.0146
1	1540 (29.98)	674 (28.83)	866 (30.95)	
≤2	485 (9.44)	193 (8.25)	292 (10.44)	
**Chewing problem**				
Comfortable	3157 (61.47)	1460 (62.45)	1697 (60.65)	0.1151
Uncomfortable	1979 (38.53)	878 (37.55)	1101 (39.35)	
**Dental visits during the past year**				
No	3777 (73.54)	1629 (69.67)	2148 (76.77)	<0.0001
Yes	1359 (26.46)	709 (30.33)	650 (23.23)	
**Self-perceived oral health**				
Good	2623 (51.07)	1195 (51.11)	1428 (51.04)	0.4822
Poor	2513 (48.93)	1143 (48.89)	1370 (48.96)	
**Present teeth, *n***	19.01 ± 0.15	18.46 ± 0.22	19.47 ± 0.19	0.0003
<20	2024 (39.41)	968 (41.40)	1056 (37.74)	0.0138
≥20	3112 (60.59)	1370 (58.60)	1742 (62.26)	
**Handgrip strength, kg**	27.56 ± 0.15	35.05 ± 0.15	21.32 ± 0.11	<0.0001
Normal, *n*	4218 (82.13)	2033 (86.95)	2185 (78.09)	<0.0001
Low	918 (17.87)	305 (13.05)	613 (21.91)	

Continuous variables are presented as the means ± standard error. Categorical variables are presented as *n* (%). * *p*-value < 0.05 using the *t*-test or chi-square test.

**Table 2:** Multiple logistic regression analysis of the association between the present teeth and low muscle strength.

Present Teeth	Model 1	Model 2	Model 3	Model 4
	OR (95% CI)	OR (95% CI)	OR (95% CI)	OR (95% CI)
<20	1.73 (1.48–2.03)	1.48 (1.25–1.74)	1.36 (1.15–1.62)	1.35 (1.13–1.61)
≥20	1.00	1.00	1.00	1.00

OR: odds ratio. 95% CI: 95% confidence interval. Model 1: unadjusted. Model 2: adjusted for sex and age. Model 3: adjusted for sex, age, education, income, smoking, drinking, exercise, BMI, and comorbidity. Model 4: adjusted for sex, age, education, income, smoking, drinking, exercise, BMI, comorbidity, brushing, oral hygiene products, chewing problem, dental visits during the past year, and self-perceived oral health.

**Table 3:** Multiple logistic regression analysis of the association between present teeth and low muscle strength by subgroup.

	Men				Women			
Present Teeth	Model 1	Model 2	Model 3	Model 4	Model 1	Model 2	Model 3	Model 4
	OR (95% CI)	OR (95% CI)	OR (95% CI)	OR (95% CI)	OR (95% CI)	OR (95% CI)	OR (95% CI)	OR (95% CI)
<20	1.96 (1.47–2.62)	1.64 (1.21–2.21)	1.54 (1.12–2.12)	1.41 (1.02–1.95)	1.71 (1.40–2.09)	1.40 (1.13–1.72)	1.29 (1.04–1.59)	1.31 (1.06–1.61)
≥20	1	1	1	1	1	1	1	1

OR: odds ratio. 95% CI: 95% confidence interval. Model 1: unadjusted. Model 2: adjusted for sex and age. Model 3: adjusted for sex, age, education, income, smoking, drinking, exercise, BMI, and comorbidity. Model 4: adjusted for sex, age, education, income, smoking, drinking, exercise, BMI, comorbidity, brushing, oral hygiene products, chewing problem, dental visits during the past year, and self-perceived oral health.

## Data Availability

The dataset analyzed for this study can be found at https://knhanes.kdca.go.kr/knhanes/eng/index.do (accessed on 16 June 2022).
